# Tissue-resolved proteomic characterization of oat grains guided by metabolite MALDI mass spectrometry imaging

**DOI:** 10.1007/s00216-026-06555-6

**Published:** 2026-05-11

**Authors:** Darren Lau, Parul Mittal, Jack Scanlan, Leigh Donnellan, Manuela Klingler-Hoffmann, Peter Hoffmann

**Affiliations:** School of Pharmacy and Biomedical Science, College of Health, Adelaide University, Adelaide, SA 5000 Australia

**Keywords:** Oat, Grain compartments, MALDI-MSI, Metabolites, Proteomics

## Abstract

The metabolic architecture of oat grains remains poorly understood at the cellular and proteome levels despite increasing interest for applications such as nutritional enhancement and functional crop improvement. In this study, we performed matrix-assisted laser desorption and ionization mass spectrometry imaging (MALDI-MSI) to map metabolite distributions across oat grain tissues and delineate major compartments of metabolite localization. These metabolite-rich regions, notably the bran, endosperm, and embryo, were then isolated for proteomic analysis, which detected around 3700 proteins across all tissue types. Gene ontology analysis highlighted specialized functions and biological processes associated with each compartment. Further interrogation of key proteins revealed the enrichment of core cellular functions such as energy generation, translation, and stress resilience in the embryo, nutrient storage in the endosperm, and an intermediate functional profile in the bran. These findings provide new insights into the spatial organization of metabolic functions within oat grains and warrant further exploration of sub-regions for a deeper understanding of the oat metabolic architecture.

**Graphical Abstract:**

**Figure:**
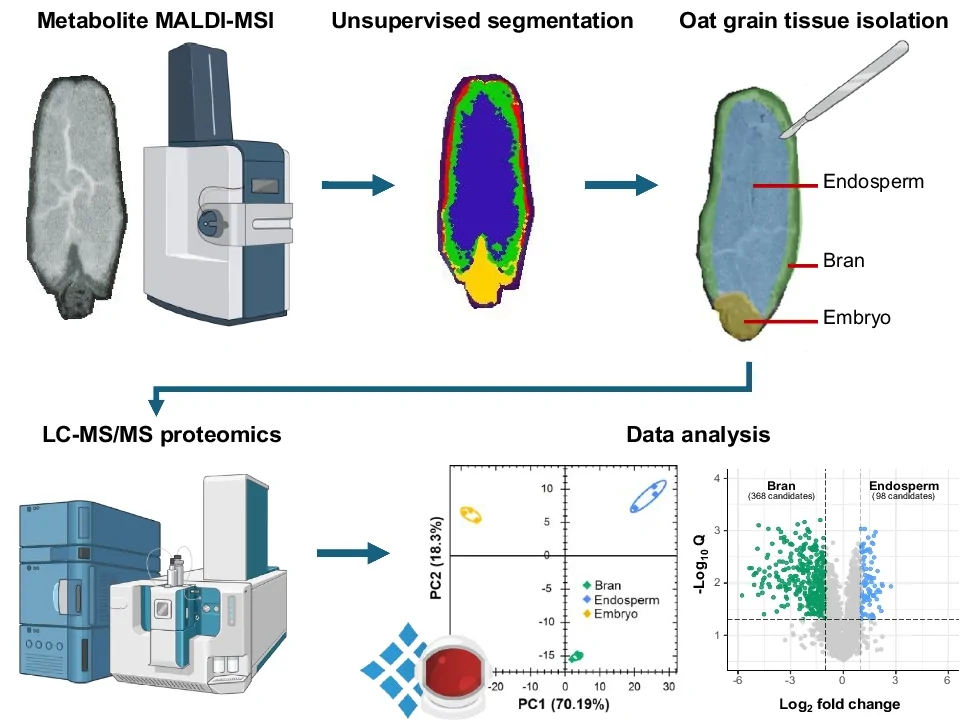

**Supplementary Information:**

The online version contains supplementary material available at https://doi.org/10.1007/s00216-026-06555-6.

## Introduction

Oat (*Avena sativa* L.) is one of the most consumed food crops and ranks seventh in global production quantity (https://www.fao.org/faostat/en/, accessed 22 January 2026). Oat possesses a distinct nutrient profile when compared to other cereal grains, characterized by its high oil and soluble fiber content [1, 2]. It also has a celiac-friendly protein profile, as avenins, which are seed storage proteins similar to gluten proteins in wheat and barley, exhibit a very low prevalence of celiac-associated immune-reactive regions [3]. Furthermore, oats are rich in avenanthramides, a group of phenolic compounds with anti-inflammatory and antioxidant properties and have been linked to weight loss, improved cardiovascular health and reduced blood cholesterol levels [4–7]. With increasing demand for healthier, more sustainable, and plant-based food sources [8], oats have gained popularity among consumers and food industries for their nutritional profile, sustainability, and low allergenicity.

Once the husk is removed, the edible portion of the oat grain, known as the groat, is primarily made up of the endosperm and embryo. The embryo is the innermost part, where the germination process is initiated, while the endosperm is the largest starchy tissue that makes up the bulk of the kernel and serves as a nutrient reservoir storage to support seedling growth and development [9]. Both compartments can be further divided into specialized tissues and cell types, with the endosperm surrounded by nutrient-rich bran (sub-aleurone, aleurone, and pericarp) layers, whereas the embryo comprises the scutellum, plumule, and radicle [10]. Although this compartmentalization is highly conserved across cereal species, the distribution of nutrients within a grain may vary [11]. For example, lipids predominantly accumulate in the embryo of barley and wheat grains but are found at higher quantities in the endosperm and aleurone layers of oat grains [12–14]. Therefore, the metabolic architecture of cereal grains is species-specific, and the insights from one cereal crop should not be applied universally.

Understanding the physiological or functional roles of each tissue type can help in tailoring genetic modification strategies for crop improvement. The overexpression of an oat endosperm-specific *WRINKLED1* transcription factor homologue has induced a nine-fold increase in oil accumulation in the endosperm of wheat grains, which typically store oil in the embryo [15]. In a separate study, the expression of a maize embryo *WRINKLED1* homologue in the maize endosperm did not result in a significant increase in oil content, highlighting tissue-specific responses to genetic engineering [16]. Proteomics has been widely used for the characterization of protein profiles to study regulatory networks in cereal grains, particularly in wheat, including studies examining different grain compartments [17, 18]. In the context of oats, proteomics has recently been performed to investigate nutrient metabolism, enabled by the availability of an annotated oat genome, providing a translated peptide sequence database [3, 19, 20]. However, these studies were largely conducted on whole grains, which may have masked tissue-specific protein regulation. To date, the knowledge on proteome variations across major oat grain compartments remains limited.

While oat grain compartments can be distinguished anatomically using conventional histological staining, such approaches primarily reflect structural and morphological characteristics rather than functional biochemical heterogeneity. Mass spectrometry imaging, particularly matrix-assisted laser desorption and ionization mass spectrometry imaging (MALDI-MSI), enables direct visualization of spatial metabolite distributions in situ, revealing metabolic compartmentalization and analyte heterogeneity that are not always evident from histological features alone. Studies applying MALDI-MSI to plant and cereal tissues have demonstrated distinct localization patterns of metabolites and lipids across tissue types, reflecting underlying biochemical processes that extend beyond visible anatomy [21].

Although MALDI–MSI following on-tissue tryptic digestion can provide spatially resolved peptide information, this approach requires enzymatic digestion and multiple sample preparation steps that can delocalize peptides and reduce spatial fidelity, particularly in starch- and lipid-rich seed tissues [14]. Furthermore, proteomic MALDI–MSI typically achieves lower proteome coverage and depth than LC–MS/MS-based bottom-up workflows, which benefit from chromatographic separation of ions and enhanced peptide identification sensitivity [22]. By contrast, metabolite-focused MALDI–MSI allows direct analysis of endogenous small molecules and lipids without enzymatic pretreatment, preserving native molecular distributions and providing robust spatial information suitable for unsupervised segmentation [23]. Using metabolite MSI to guide region delineation thus offers a scientifically rigorous, data-driven framework for defining functionally relevant tissue domains, which can then be precisely excised for in-depth, quantitative LC–MS/MS proteomic analysis. This integrated workflow leverages the complementary strengths of metabolite MSI and LC–MS/MS proteomics, linking intrinsic metabolic organization with comprehensive protein characterization to achieve a tissue-resolved understanding of oat grain biology.

Using this metabolite-guided approach, we combined MALDI–MSI with LC–MS/MS to study the protein composition of spatially defined regions within the oat grain. Metabolite MSI allowed us to identify areas with distinct molecular patterns, from which representative regions corresponding to the bran, endosperm, and embryo were selected for detailed proteomic analyses. Although the oat grain contains multiple anatomical layers, this strategy enabled us to examine key regions that reflect underlying metabolic organization. Our results show that the oat grain is metabolically heterogeneous rather than uniform. This spatial variation provides a stronger basis for future high-resolution studies and may support breeding strategies aimed at improving grain composition and quality.

## Materials and methods

### Plant materials and chemicals

Mature oat grains were provided by the South Australian Research and Development Institute (SARDI). Gelatine (#G2500), dithiothreitol (DTT), ammonium bicarbonate, Tris, sodium chloride (NaCl), urea, 2-chloroacetamide, and sodium dodecyl sulfate (SDS) were purchased from Merck, Australia. Matrix α-cyano-4-hydroxycinnanic acid (HCCA, #8201344) was acquired from Bruker Daltonics, Germany. Trypsin Gold (#V5280) was purchased from Promega, Australia. LC–MS grade formic acid and acetonitrile (ACN) were purchased from ChemSupply, Australia.

### Oat tissue sectioning

The sectioning of oat grains was performed according to a previously established protocol with minor modifications [14]. Oat grains were embedded in 10% gelatine (*w/v*) and cryo-sectioned at 20-µm thick using a cryostat set to − 20 °C (Leica, Germany). Sections were mounted onto an indium tin oxide (ITO)-coated slide (Bruker Daltonics, Germany) and stored at − 80 °C for MALDI-MSI experiments in biological duplicates or polyethylene naphthalate (PEN) membrane slides (Leica, Germany) for proteomic analysis in biological triplicates.

### Matrix-assisted laser desorption and ionization mass spectrometry imaging (MALDI-MSI)

Slides were thawed under vacuum for 30 min before 10 mg/mL HCCA, prepared in 50% ACN (*w/v*) supplemented with 100 mM NaCl, was deposited onto oat sections using the iMatrixSpray system (Switzerland) according to previously reported settings [20]. Sodium doping was performed to improve the detection of neutral lipids and metabolites [14]. The acquisition of MALDI-MSI data was performed on a timsTOF fleX mass spectrometer (Bruker Daltonics) operated through timsControl (version 3.0.21, Bruker Daltonics) and FlexImaging (version 7.0, Bruker Daltonics) in positive ionization mode at 20 µm resolution with instrument settings detailed in Table S1.

Ion distribution images were generated using SCiLS Lab (version 2023a Pro, SCiLS, Bruker Daltonics). Data were normalized to the 99% quartile using root mean square, with weak de-noising applied. MALDI-MSI datasets processed in SCiLS Lab were exported and analyzed in METASPACE (https://metaspace2020.org) [24]. The CoreMetabolome database was selected for *m/z* feature annotation, with a mass tolerance of 3 ppm and a false discovery rate (FDR) of less than 10%. The dataset was further curated to remove metabolites that are not canonically present in plants.

### Sample preparation for proteomic analysis

Compartments (bran, endosperm, and embryo) of each biological replicate identified through unsupervised segmentation of MALDI-MSI data were excised using a scalpel under a magnifying glass and transferred into a 0.5-mL Protein LoBind Tube (Eppendorf, Australia). Samples were processed for proteomic analysis on the day of tissue preparation. For protein extraction, 100 µL of 4% SDS supplemented with 100 mM Tris and 1 mM DTT was added to the excised sections. The mixture was vortexed and boiled for 5 min at 95 °C, followed by a 15-min sonication. The samples were then boiled for another 15 min and centrifuged at 20,000 × *g* for 10 min. The supernatant was collected and proteins were precipitated overnight at −20 °C using 4:1 volume of freezer-cold acetone. The supernatant was discarded after centrifugation, and protein pellet was redissolved in 6 µL of 8 M urea supplemented with 50 mM ammonium bicarbonate. Protein concentration was estimated using the Bradford assay (Bio-Rad, USA). Reduction and alkylation were performed on 10 µg protein extract via the addition of DTT (final concentration of 110 mM) for 1 h, followed by 2-chloroacetamide (final concentration of 125 mM) for 30 min. Samples were diluted to 1.6 M urea by the addition of 50 mM ammonium bicarbonate and incubated overnight with 200 ng Trypsin Gold at 37 °C. To inhibit further reaction, pH was adjusted to 2 using formic acid, and peptide clean-up was performed using C18 ZipTip® Pipette Tips (Merck, Australia), followed by drying in a vacuum centrifuge at 50 °C. Peptides were reconstituted in 20 µL 0.1% formic acid in water for LC–MS/MS analysis.

### Liquid chromatography tandem mass spectrometry (LC–MS/MS) analysis

LC–MS/MS analysis of peptides was carried out on an ACQUITY UPLC M-Class system (Waters, USA) coupled with a ZenoTOF 7600 mass spectrometer (SCIEX, Singapore). Raw data were acquired using SCIEX OS (version 3.1.0). Peptides (equivalent to 500 ng) were loaded at 2.4 µL/min onto a 1.5 µm 100Å Reprosil Saphir C18 200µm ID × 150 mm (#AR1-45143, PremierLCMS, Canada) column heated to 40 °C. Peptides were separated over a gradient: 3–25% mobile phase B (0.1% formic acid in ACN) over 40 min, followed by a washing step of 90% mobile phase B for 3 min and an equilibration of 3% mobile phase B for 7 min. A Zeno SWATH acquisition scheme (64 variable-size windows covering a 400–750 *m*/*z* precursor mass range) was performed with 13 ms MS/MS accumulation time. Spray voltage was set at 5000 V, ion source gases 1 and 2 at 20 and 15 psi, respectively, curtain gas at 35 psi, collision gas (CAD) at 7 psi, and source temperature at 150 °C.

### Proteomics data processing, analysis, and visualization

Raw LC–MS/MS data was analyzed using Spectronaut® (version 19.7.250203, Biognosys, Switzerland) with the directDIA+™ workflow and default parameters (enzyme, trypsin/P; fixed modification, carbamidomethyl (C); variable modifications, acetyl (protein N-terminus) and oxidation (M); false discovery rates, 1%). MS/MS data were searched against an in-house FASTA database comprising predicted protein sequences derived from gene annotations of the *Avena sativa* – OT3098 v2 genome assembly (PepsiCo, https://wheat.pw.usda.gov/jb?data=/ggds/oat-ot3098v2-pepsico) containing 257,893 entries. Differentially abundant proteins were considered using MS2 level quantities only, a log_2_ fold change cut-off of 1, and an adjusted *p*-value threshold of less than 0.05. Fold-changes were visualized with volcano plots generated in RStudio. Principal component analyses (PCA) were performed and visualized with Spectronaut®, and functional enrichment analysis was performed on gProfiler, selecting *Avena sativa* OT3098 as the reference organism. Heatmaps were generated using MetaboAnalyst 6.0 or Heatmapper2 [25]. Bar graphs and dot plots were generated using GraphPad Prism (version 10.0.2, Boston, USA), with statistical significance assessed by ANOVA.

## Results

### MALDI-MSI of oat grains reveals main compartments of metabolite accumulation

**Fig. 1:**
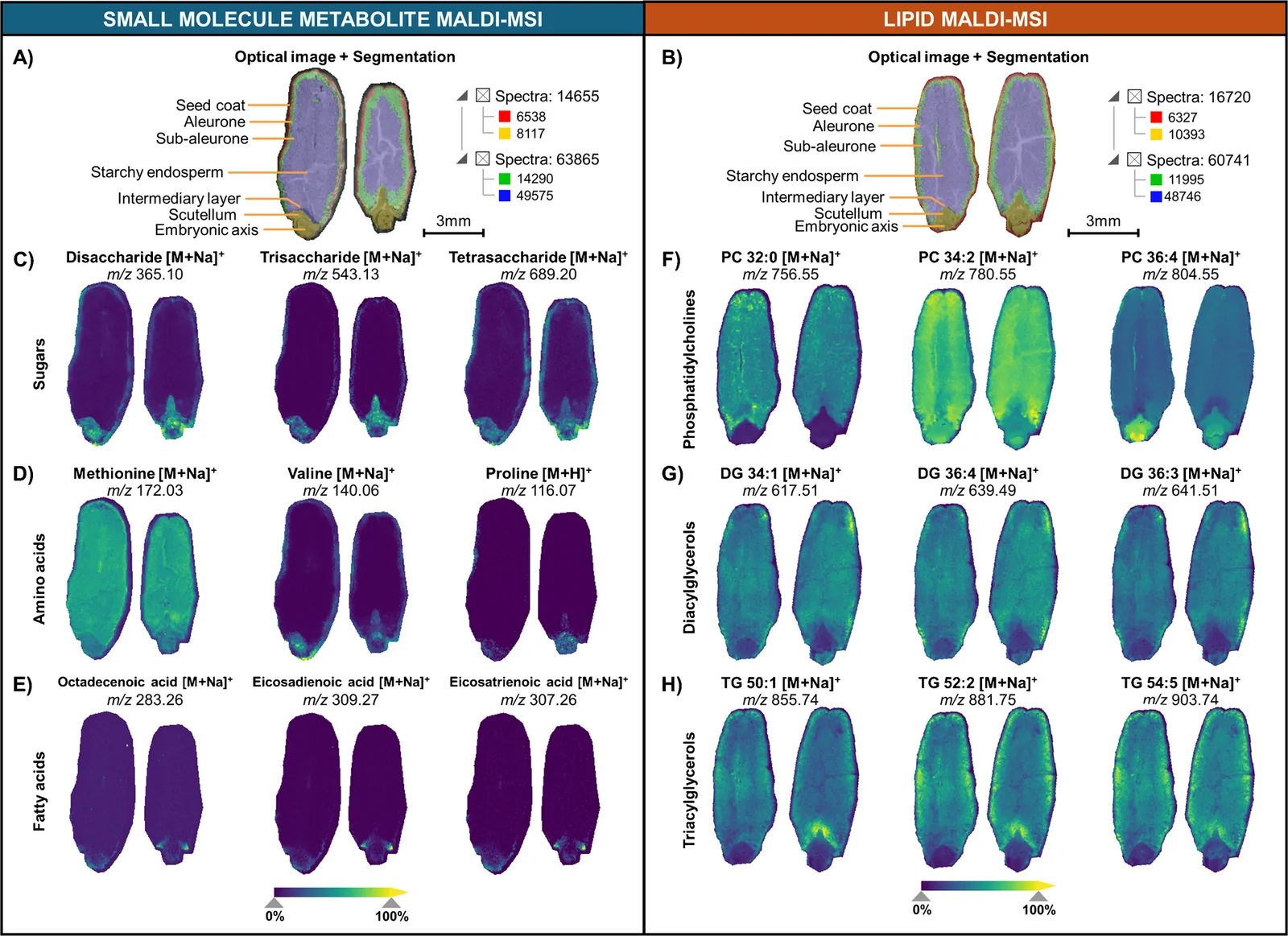
Metabolite MALDI-MSI of oat grain tissue. Optical image of oat sections used for **A** small molecule metabolite and **B** lipid MALDI-MSI analysis, overlayed with unsupervised segmentation maps. Seed coat, aleurone, and sub-aleurone are classified as bran layers. Ion distribution images were generated for sodiated adducts of **C** sugars, **D** amino acids, **E** fatty acids, **F** phosphatidylcholines, **G** diacylglycerols, and **H** triacylglycerols, tentatively annotated using METASPACE. Intensity ranged from low (blue) to high (yellow)

To identify major compartments in oat grains for manual excision prior to proteomic analysis, MALDI-MSI was first performed to visualize small molecule metabolite (Fig. 1A) and lipid (Fig. 1B) distributions across oat tissue sections. Unsupervised segmentation of MALDI-MSI data highlighted distinct regions characterized by unique metabolite profiles, mainly corresponding to the seed coat, aleurone layers, endosperm, and embryo (Fig. 1A, B). Additional segmentation revealed finer structural heterogeneity within the outer grain layers, with a similar trend observed across both datasets (Fig. S1). MALDI-MSI analyses yielded 93 tentatively annotated metabolites and lipids (Table S2).

Ions at *m/z* 365.10, 543.13, and 689.20, annotated as sodiated adducts of saccharide sugars, were localized predominantly in the seed coat and embryo (Fig. 1C). For amino acids, methionine was detected ubiquitously across the whole section, while valine [M + Na]^+^, *m/z* 140.06, and proline [M + H]^+^, *m/z* 116.07, showed similar distribution patterns as the sugars (Fig. 1D). Free fatty acids, such as octadecenoic acid (*m/z* 283.26), eicosadienoic acid (*m/z* 309.27), and eicosatrienoic acid (*m/z* 307.26) were found only in the embryo, particularly in the scutellum (Fig. 1E). Similar to previous reports [20], phosphatidylcholines (*m/z* 756.55, 780.55, and 805.44) were observed in all regions of the grain, with each species displaying distinct accumulation patterns (Fig. 1F). Glycerolipids, such as diacylglycerols (*m/z* 617.51, 639.49, and 641.51) and triacylglycerols (*m/z* 855.74, 881.75, and 903.74), were enriched in the endosperm, with the latter showing stronger signals in the aleurone layers (Fig. 1G, H).

### Proteome profiling of oat grain compartments

**Fig. 2:**
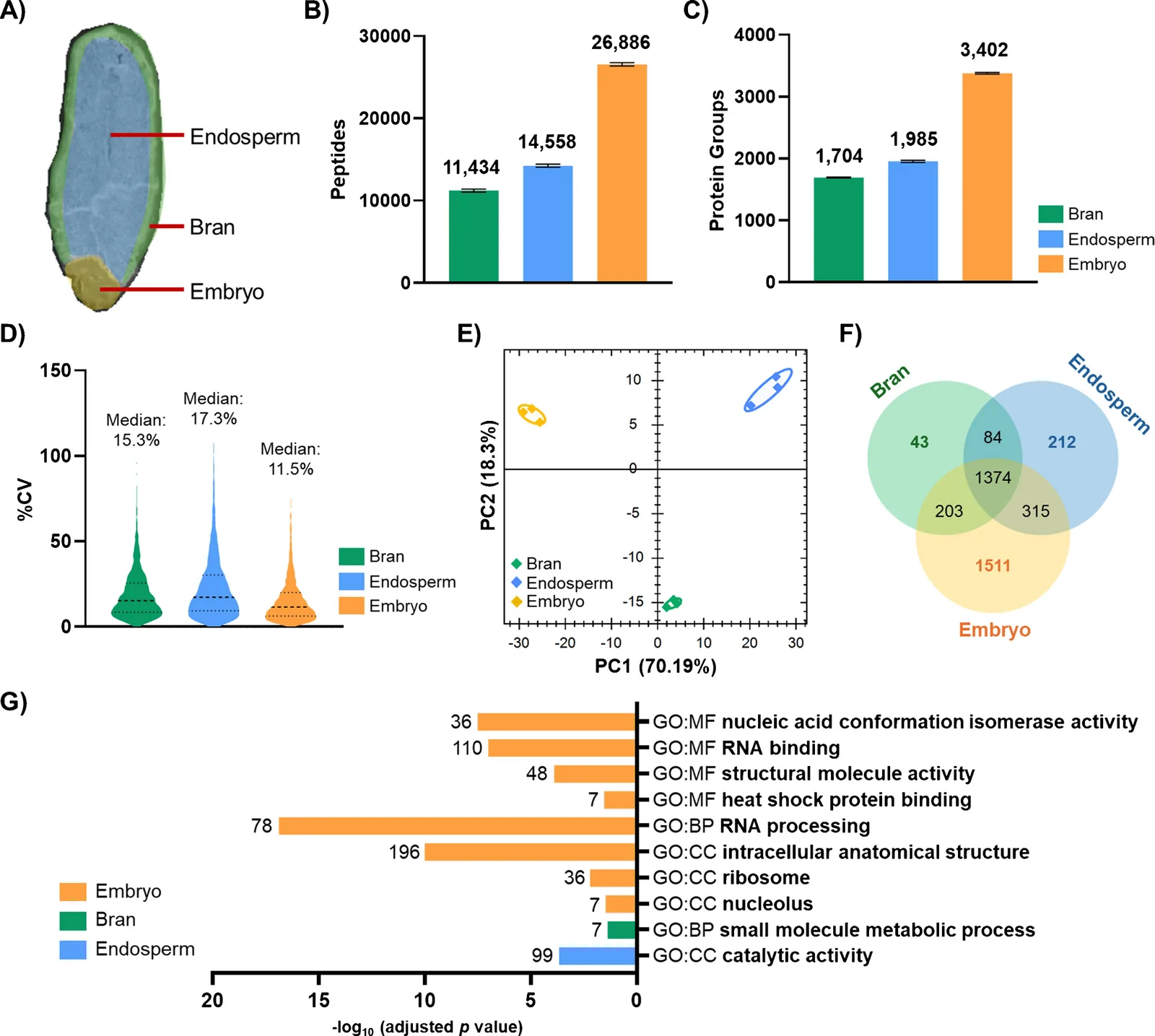
Proteomic analysis of oat grain compartments. **A** The bran, endosperm, and embryo regions isolated for proteomic analysis. **B** Number of peptides and **C** protein groups identified. **D** Violin plot illustrating %CV of identified protein groups in each tissue type. **E** PCA plot for bran, embryo, and endosperm proteomes. **F** Venn diagram highlighting the overlap of protein group identifications across tissue types. **G** Functional enrichment analysis of proteins uniquely identified in each tissue type

Based on the MALDI-MSI data, we selected three regions of interest for proteomic analysis, namely the endosperm, bran, and embryo (Fig. 2A), as technical limitations prevented further isolation of individual bran components (pericarp, seed coat, aleurone, and sub-aleurone). Proteomic analysis identified 11,434 peptides in the bran and 14,558 in the endosperm, with the highest number observed in the embryo at 26,886 (Fig. 2B). This corresponded to 1704, 1985, and 3402 protein groups in the bran, endosperm, and embryo, respectively (Fig. 2C). The median coefficient of variation in percentage (% CV) across all three tissue types ranged from 11.5% to 17.3%, indicating quantitative reproducibility (Fig. 2D). PCA revealed clear separation of bran, endosperm, and embryo fractions, with the first principal component explaining up to 70% of variations in their proteome profiles (Fig. 2E). Across the three different tissue types, a total of 3742 distinct protein groups were identified, in which 43 were unique to the bran, 212 to the endosperm, and 1511 to the embryo (Fig. 2F). Gene ontology analysis revealed the enrichment of proteins associated with translational activity in the embryo, small molecule metabolism in the bran, and catalytic activity in the endosperm (Fig. 2G).

### Differential abundance analysis of proteins identified across three tissue types

**Fig. 3:**
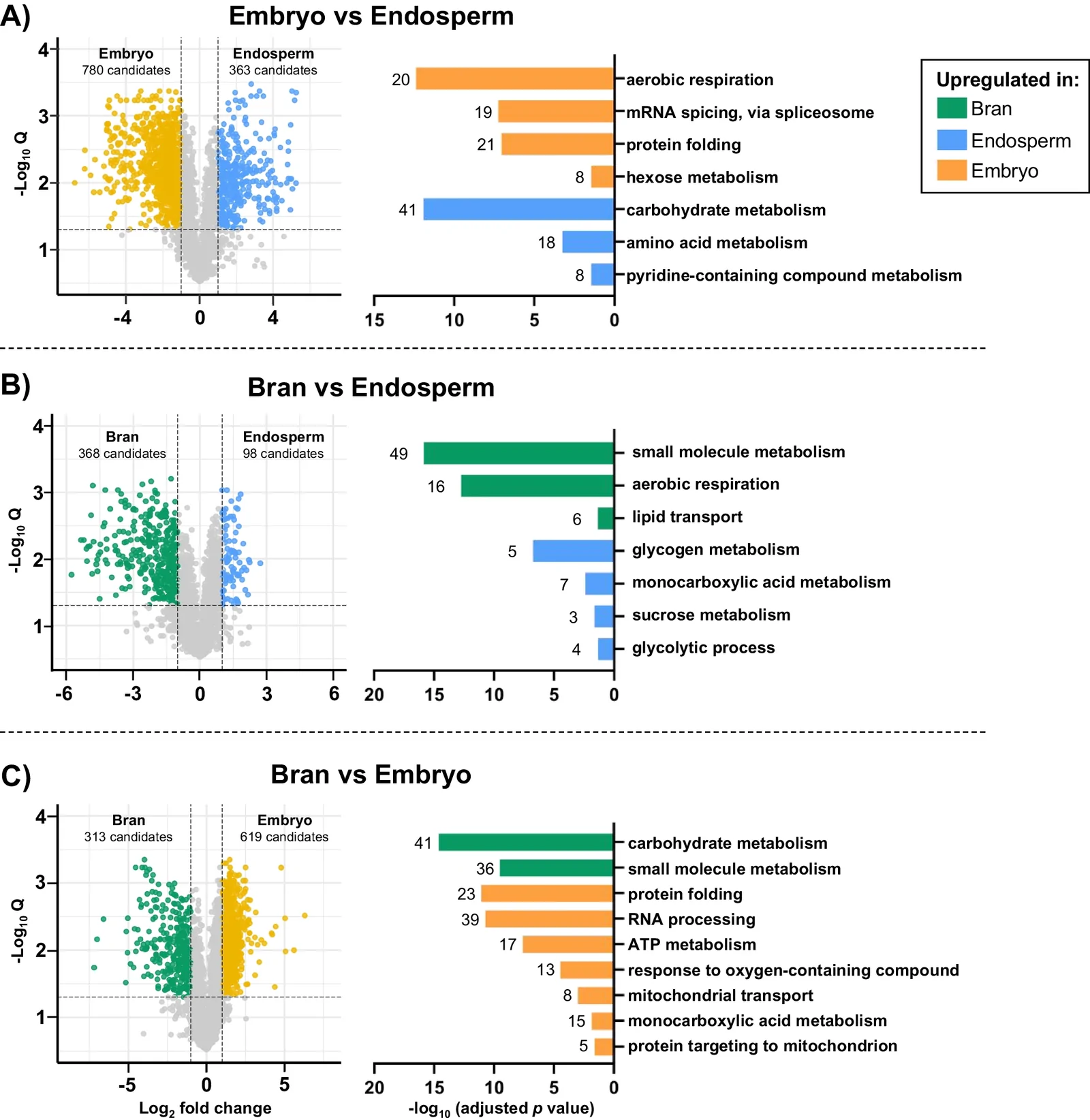
Differential abundance analysis of proteins identified across three tissue types. Volcano plots (*left*) and bar graphs showing biological processes enriched via gene ontology (*right*) for proteins that were differentially abundant in **A** embryo vs endosperm, **B** bran vs endosperm, and **C** bran vs embryo

Next, we further interrogated proteome variations across the oat bran, endosperm, and embryo with differential abundance analysis, with candidates considered using a fold change cut-off of at least 2 (log_2_ ≥ 1). Comparison between embryo and endosperm revealed that 780 proteins were upregulated in the embryo, while 363 were upregulated in the endosperm (Fig. 3A). Proteins upregulated in the embryo were mainly associated with energy generation and translation, while those upregulated in the endosperm were involved in carbohydrate and amino acid metabolism (Fig. 3A). In the bran vs endosperm comparison, a total of 466 candidates were identified, with 368 upregulated in the bran and 98 in the endosperm (Fig. 3B). Terms related to small molecule metabolism and aerobic respiration were enriched with upregulated proteins in the bran, while those in the endosperm were related to carbohydrate regulation such as glycogen (or glucan) metabolism, sucrose metabolism, and glycolysis (Fig. 3B). Finally, comparison of the bran and embryo identified 313 proteins upregulated in the bran that were associated with carbohydrate and small molecule metabolism, and 619 proteins upregulated in the embryo that were related to RNA processing and energy generation (Fig. 3C).

### Differential abundances of proteins involved in core cellular functions

**Fig. 4:**
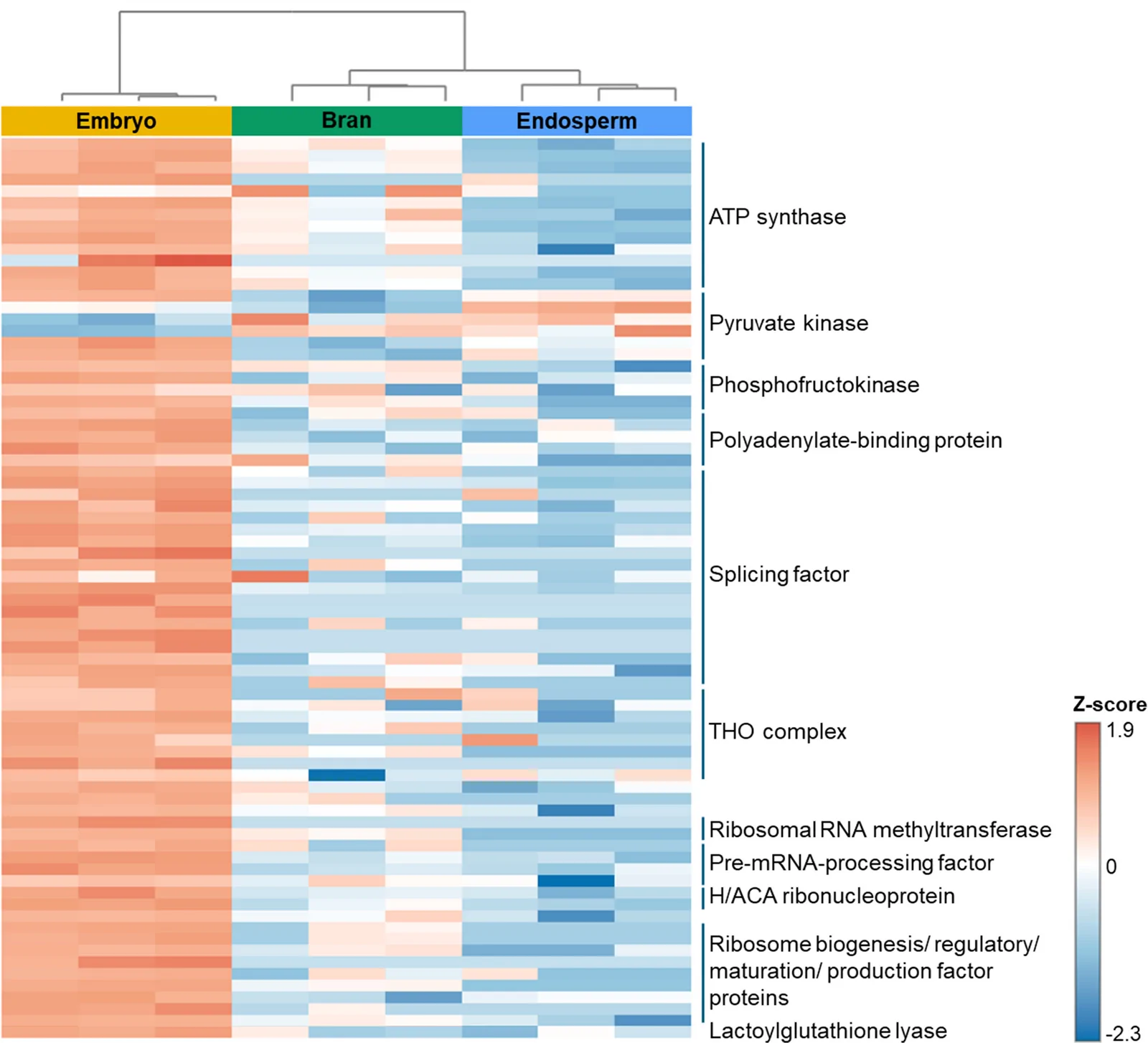
Proteins involved in core cellular functions across oat bran, endosperm, and embryo tissues. Heatmap displaying the abundances of proteins (log_2_-transformed and *Z*-score normalized) associated with ATP synthesis, translation capacity, and protective roles

Differential abundance analyses highlighted enrichment of proteins involved in energy generation and translational processes in the embryo across multiple tissue comparisons. To resolve these patterns, we investigated tissue-specific expression of these proteins and their subunits or isoforms (Fig. 4). Proteins associated with energy metabolism, such as ATP synthase, pyruvate kinase, and phosphofructokinase, generally showed elevated abundances in the embryo, with intermediate levels in the bran. Isoform-specific abundance patterns were observed for pyruvate kinase, as distinct isoforms showed differential abundance across three tissue types. Similar abundance patterns were observed for proteins involved in eukaryotic translation machinery, including polyadenylate-binding proteins, splicing factors, pre-mRNA processing factors, ribosomal RNA methyltransferases, and proteins associated with ribosome biogenesis and production. The elevation of these proteins suggests enhanced translational capacity in the embryo. In addition, lactoylglutathione lyase, an enzyme involved in the detoxification of reactive carbonyl species, was more abundant in the embryo, in alignment with the role of the embryo as a metabolically active region.

### Differential abundances of proteins involved in nutrient synthesis and storage

**Fig. 5:**
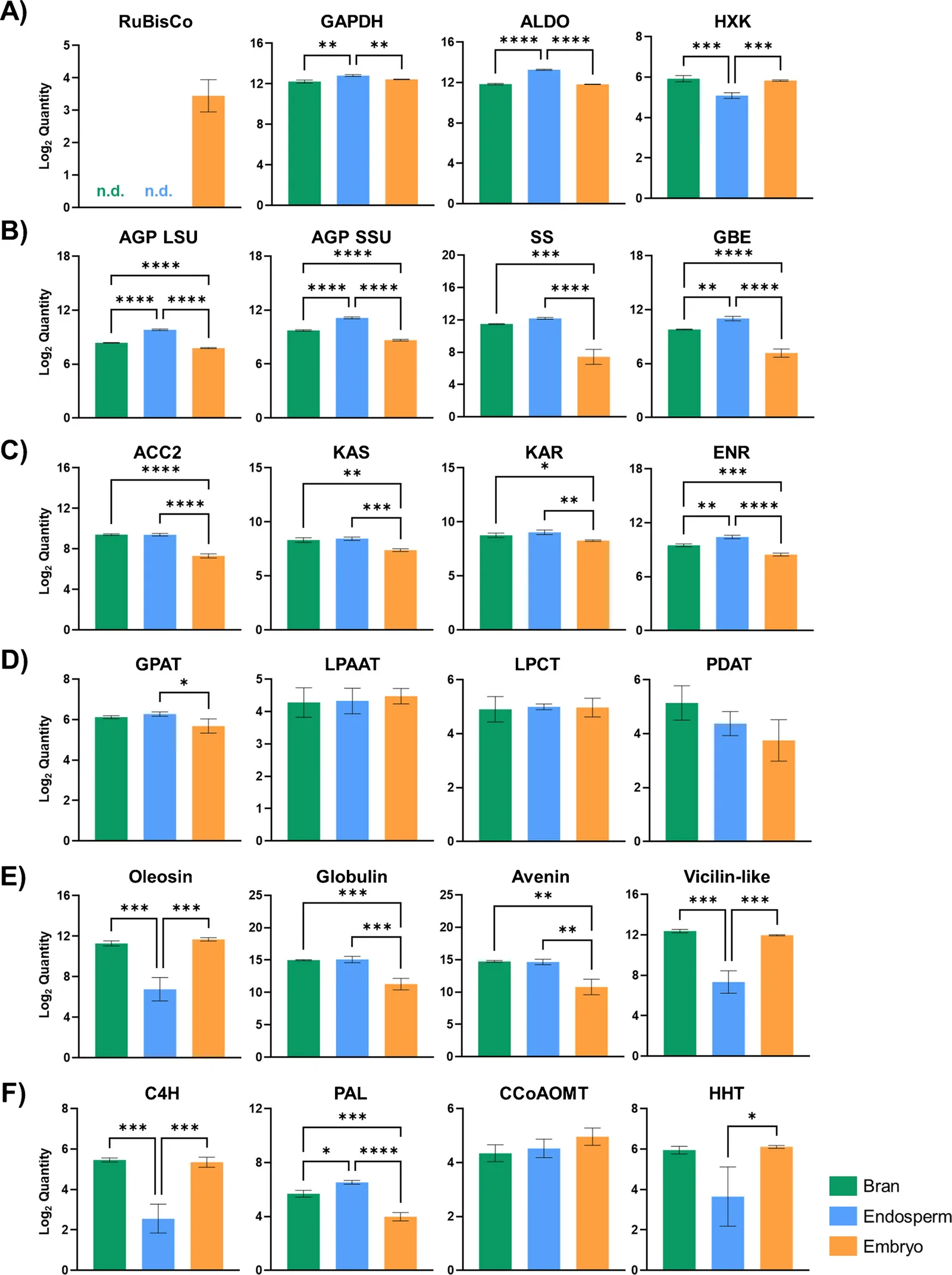
Abundances of proteins involved in synthesis and storage. Bar graphs showing abundances (expressed in log_2_ quantity) of proteins involved in **A** photosynthesis, **B** starch synthesis, **C** fatty acid synthesis, **D** triacylglycerol assembly, **E** structural function and protein storage, and **F** avenanthramide synthesis. n.d., not detected. *p < 0.05; **p < 0.01; ***p < 0.005; ****p < 0.001

We next examined the abundances of proteins that were associated with key crop quantitative traits, such as starch, oil, and protein content, to further understand the coordination of metabolic output. The carbon-fixing enzyme, ribulose-1,5-bisphosphate carboxylase/oxygenase (RuBisCo), was detected exclusively in the embryo. Other photosynthetic enzymes, glyceraldehyde-3-phosphate dehydrogenase (GAPDH) and aldolase (ALDO), were significantly more abundant in the endosperm compared to both the bran and embryo. In contrast, hexokinase (HXK), which phosphorylates glucose substrates into glucose-6-phosphate for starch or oil synthesis, was less abundant in the endosperm (Fig. 5A). Starch synthesis enzymes, such as glucose-1-phosphate adenylyltransferase large (AGP LSU) and small subunits (AGP SSU), starch synthase (SS), and glucan-branching enzyme (GBE), were most abundant in the endosperm, followed by bran and embryo (Fig. 5B). A similar trend was observed for enzymes involved in fatty acid synthesis, including acetyl-CoA carboxylase 2 (ACC2), 3-oxoacyl-acyl-carrier-protein (ACP) synthase (KAS), 3-ketoacyl-ACP reductase (KAR), and enoyl-ACP reductase (ENR) (Fig. 5C). Enzymes involved in downstream acyl modification and triacylglycerol assembly did not show significant differences across the compartments, except for glycerol-3-phosphate acyltransferase (GPAT), which was significantly lower in the embryo (Fig. 5D). In terms of seed structural and storage proteins, oleosins and vicilin-like proteins presented the lowest abundances in the endosperm, while globulins and avenins were significantly more abundant in the bran and endosperm as compared to the embryo (Fig. 5E). We also investigated the tissue-specific abundances of enzymes in the avenanthramide synthesis pathway. Cinnamic acid 4-hydroxylase (C4H) and phenylalanine ammonia lyase (PAL) displayed contrasting patterns, with C4H being least abundant in the endosperm, whereas PAL was most highly expressed in this compartment (Fig. 5F). Hydroxycinnamoyl CoA:hydroxyanthranilate N-hydroxycinnamoyl transferase (HHT), the key avenanthramide synthesis enzyme in oats, was significantly lower abundant in the endosperm in relative to the bran and embryo (Fig. 5F).

**Fig. 6:**
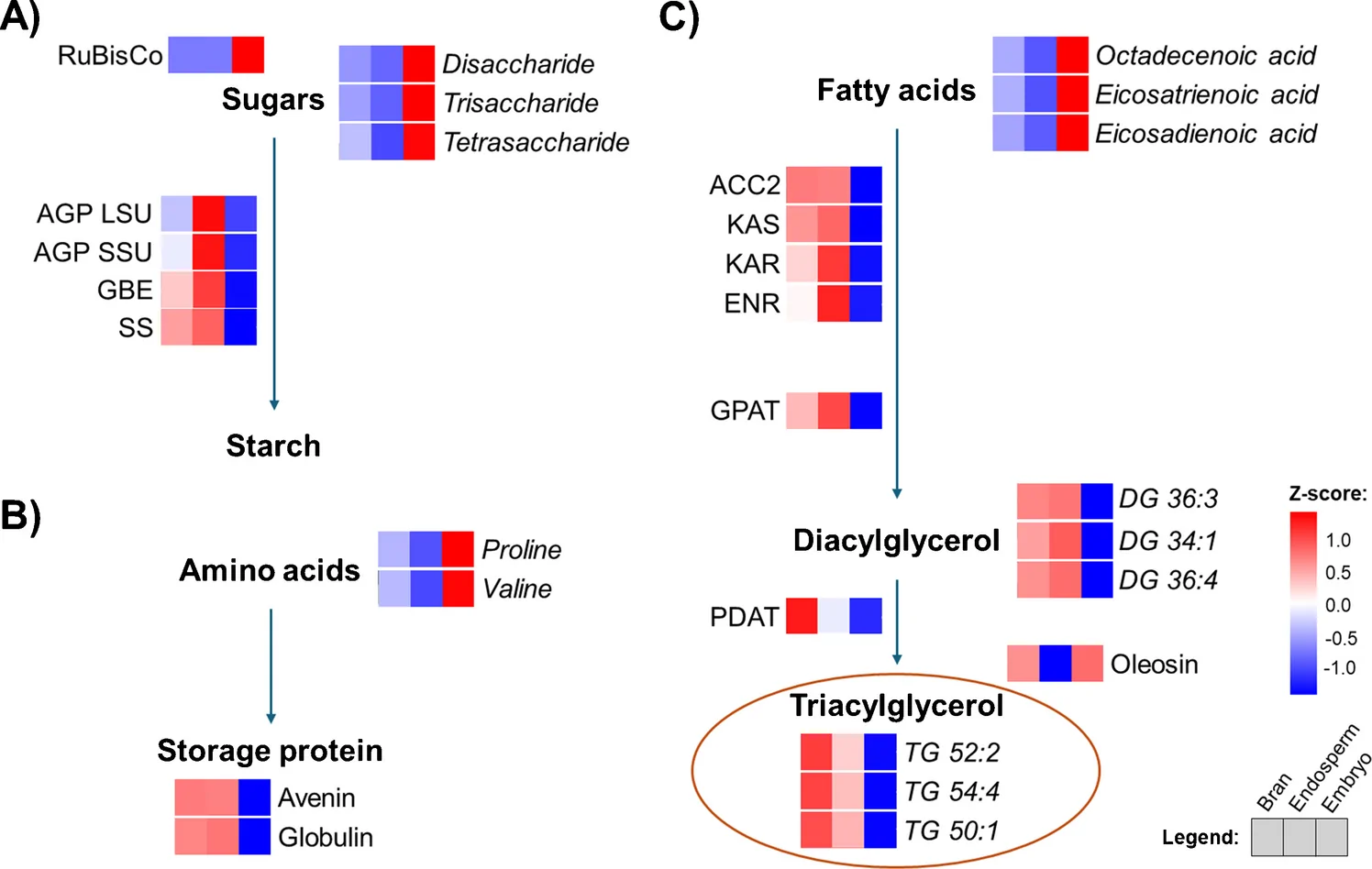
Pathway overview of macronutrient biosynthesis across bran, endosperm, and embryo tissues. Heatmaps illustrate the relative abundances of proteins and metabolites (*italicized*) associated with **A** starch, **B** protein, and **C** oil synthesis pathways. Values represent mean abundances (proteins) or median intensities (metabolites) and are *Z*-score normalized

As differential abundances of proteins involved in macronutrient synthesis were observed across the three tissue types, we investigated their correspondence with MALDI-MSI-derived metabolite distributions. The accumulation of sugars in the embryo was consistent with the exclusive detection of RuBisCO in this compartment. In contrast, the abundances of starch biosynthetic enzymes showed a negative association with sugar-related metabolites (Fig. 6A). Amino acids, the building blocks of proteins, were also enriched in the embryo and exhibited a negative association with the localization of storage proteins (Fig. 6B). In lipid metabolism, the accumulation of diacylglycerols and triacylglycerols in the bran and endosperm positively correlated with the higher abundance of fatty acid synthesis enzymes in these tissues. Similarly to sugars and amino acids, fatty acids were enriched in the embryo (Fig. 6C). These results support the roles of the endosperm and bran as primary macronutrient reservoirs, while the embryo accumulates metabolically accessible molecules.

## Discussion

Previous studies have employed untargeted MALDI-MSI of metabolites, lipids, or peptides to define regions of interest within mammalian or plant tissues for subsequent proteomic experiments [26–34], offering a spatial biological context for deeper molecular interrogation. To date, this approach has not been applied to cereal grains, which have highly compartmentalized tissues with cell types adapted to specialized functions [35, 36]. In this study, we first investigated metabolic signatures across oat grain tissue via an untargeted MALDI-MSI approach to segment regions of interest for subsequent proteomic analyses. Marked differences in protein composition were revealed between bran, endosperm, and embryo tissues. Our findings consistently highlight the embryo as a developmentally active compartment with enhanced roles in energy metabolism, translation capacity, and protective mechanisms, the endosperm as a storage reservoir, and the bran as an intermediate tissue with both metabolic and storage purposes. We interrogated proteins and enzymes that underpin crop quality traits, particularly those associated with nutrient content and allocation.

Oats are characterized by a relatively high oil content, which has attracted considerable research interest due to its impact on grain processing and end-use quality [37–39]. Free fatty acids were detected exclusively in the scutellum. In contrast, elevated levels of ACC2 and downstream fatty acid synthesis enzymes indicate high regulation of de novo lipid synthesis in the endosperm, suggesting differential partitioning of lipid intermediates across tissues. LPAAT and LPCT, both involved in phospholipid biosynthesis, were uniformly abundant across all tissue types, consistent with the widespread detection of phospholipids in bran, endosperm, and embryo. Notably, the isolation of the bran layer as a distinct compartment revealed significant tissue-specific differences in GPAT abundances, a pattern not observed in previous studies comparing only endosperm and embryo fractions [20]. PDAT, which catalyses the formation of triacylglycerols by the transfer of an acyl chain from phosphatidylcholines to diacylglycerols, is the least abundant in the embryo, further corroborating the higher rate of phospholipid turnover in the bran and endosperm into oil formation [40]. Oleosins, which stabilize assembled triacylglycerols in the form of oil bodies, were least abundant in the endosperm, consistent with reports that oil bodies tend to fuse into larger structures in oats, and this would be hindered by high oleosin abundance [41]. The higher abundance of oleosins in the bran may contribute to enhanced stabilization and retention of oil within the aleurone layer, in agreement with the elevated triacylglycerol signals observed in our MALDI-MSI data. These observations underline the importance of higher-resolution, tissue-resolved analyses for accurately interpreting pathway regulation.

Key photosynthetic enzymes, including RuBisCo, GAPDH, and ALDO, are generally downregulated during grain development as the requirement for de novo carbon fixation decreases and metabolism shifts toward nutrient accumulation [19]. Interestingly, while we still observed elevated abundances of GAPDH and ALDO in the endosperm as compared to the other compartments, RuBisCo, which is the primary carbon-fixing enzyme, was not detected in this tissue. We hypothesize that this could be due to the endosperm no longer carrying out active carbon fixation as seeds mature, while the presence of RuBisCo in the embryo after maturation likely helps support rapid metabolic activation during seed germination [42]. This is further supported by the accumulation of sugars in the embryo as demonstrated using MALDI-MSI. In contrast, the lower abundance of hexokinase, along with the high abundances of starch biosynthesis enzymes in the endosperm, suggests a metabolic bias toward starch accumulation rather than glycolytic flux [35]. On the other hand, major seed storage proteins, such as globulins and avenins, were found in higher abundances in the bran and endosperm. In contrast, amino acids, as fundamental building blocks of proteins, were predominantly enriched in the embryo, followed by the bran. These observations further reinforce the role of the endosperm as the primary storage tissue with high synthetic capacity, while highlighting the embryo as a metabolically active compartment rather than a site of macromolecular storage.

By referencing our findings to wheat, a model cereal in which the proteome and metabolome have been extensively characterized in the past, we identified several shared molecular features. For example, MALDI-MSI demonstrated that methionine accumulates throughout the entire wheat section, valine in the embryo, and sugars in both embryo and bran, which are consistent with our observations in oats [43]. Research on the wheat metabolome at cell-type resolution demonstrated a decline in sugars and amino acids in the starchy endosperm, but greater accumulation in the aleurone throughout grain development [18]. This corresponds to the greater composition of gluten proteins in the endosperm as compared to the aleurone in mature wheat grains, as reported in a separate study [17]. Isolation of sub-aleurone layers has revealed distinct proteomic profiles compared to both aleurone and endosperm tissues, highlighting complex molecular heterogeneity within cereal grain architecture [17, 44]. From our MALDI-MSI analyses, major segments corresponding to the seed coat, aleurone/sub-aleurone layers, endosperm, and the embryo were delineated. Notably, additional differences were observed within these major regions, indicating further sub-regional specialization. While isolating on millimeter-scale may introduce cross-contamination between the compartments, the observed proteomic differences between regions indicate that this approach is sufficient to capture broad molecular heterogeneity across major grain compartments. As evidenced in this study, the expression of specific protein isoforms may be tissue-dependent, and understanding their spatial distribution is critical to inform optimal breeding strategies for crop improvement. However, finer spatial resolution will be required to resolve cell-type-specific proteomic variation. Nevertheless, future integration of laser capture microdissection may enable targeted isolation of specific cell types for more refined spatially resolved proteome profiling of oat grains [45].

In addition to macronutrients, oats also contain a unique group of phenolic alkaloids known as avenanthramides, which are often used in cosmetic products due to their anti-inflammatory, antioxidant, and antipruritic properties [6, 46]. Here, we demonstrated tissue-specific abundances of enzymes involved in avenanthramide synthesis. While C4H and PAL contribute to avenanthramide biosynthesis, they also participate broadly in flavonoid biosynthetic pathways [47]; hence, their abundances may be directly indicative of avenanthramide accumulation. On the other hand, HHT serves as a more specific marker of avenanthramide production since it catalyzes the committed step in avenanthramide biosynthesis [48]. We observed a reduced abundance of HHT in the endosperm as compared to the bran and embryo, which corroborates their accumulation in the outer grain layers and involvement in oat defense mechanisms against biotic stress [49, 50]. Targeted MALDI-MSI experiments to visualize the accumulation of avenanthramide may be established in the future to further understand the molecular regulation of these unique compounds.

Overall, this study provides a proof-of-concept for the integration of spatial metabolomics to define biologically relevant regions with tissue-resolved proteomics for the biological characterization of cereal grains. We demonstrate that oat grain compartments are characterized by distinct protein profiles that reflect their specialized biological roles. This work establishes a framework for understanding how metabolic capacity and nutrient allocation are spatially organized within cereal grains, with implications for crop improvement strategies aimed at modifying grain composition and resilience against stress. Importantly, these observations reflect differences in protein abundance and should be interpreted as correlative rather than indicative of metabolic flux or pathway activity. While patterns such as RuBisCo enrichment in the embryo or variation in hexokinase abundance suggest potential functional specialization, no direct conclusions regarding metabolic activity can be drawn without complementary metabolomic or flux balance analyses. Several avenues for future improvement can be pursued. We recommend incorporating laser capture microdissection to isolate tissues for interrogation at cell-type resolution. This approach should also be extended to metabolically active developing grains to enhance insights into dynamic pathway regulation. Feature annotation could also be improved by incorporating MS/MS validation or ion mobility separation to resolve isobaric or isomeric molecules [14]. In addition, integrating metabolic flux analyses would help validate metabolite accumulation and strengthen links between proteomic changes and metabolic outcomes in oat grains.

## Conclusion

Oat grains are highly compartmentalized seeds with each tissue fulfilling a distinct biological role. This is demonstrated using metabolite MALDI-MSI, which classifies ion signals into distinct regions. Subsequent proteomic analysis of these regions revealed clear functional specialization, with core cellular functions dominating in the embryo to support energy generation, translation, and protection against stress, while nutrient synthesis and storage pathways are preferentially enriched in the endosperm and bran. These findings highlight the necessity of tissue-specific proteomic analyses when investigating particular pathways or proteins of interest, as bulk grain proteomics may obscure compartment-specific regulation. This study provides proof of concept for future studies to explore the metabolic architecture of cereal grains at cell-type resolution to open new avenues for crop improvement.

## Supplementary Information

Below is the link to the electronic supplementary material.

Supplementary file1 (DOCX 552 KB)

## Data Availability

Proteomics data were deposited to the ProteomeXchange Consortium via the PRIDE partner repository with the dataset identifier PXD074044.
