# Supplementary material for: Tissue-resolved proteomic characterization of oat grains guided by metabolite MALDI mass spectrometry imaging
Source: Anal Bioanal Chem. 2026 May 11;418(20):6619–32. doi: 10.1007/s00216-026-06555-6 (PMC13619812; doi:10.1007/s00216-026-06555-6)
Supplement: Supplementary file 1 [file 216_2026_6555_MOESM1_ESM.docx]

**SUPPLEMENTARY INFORMATION**

**Tissue-resolved proteomic characterisation of oat grains guided by metabolite MALDI mass spectrometry imaging**

Darren Lau^1^, Parul Mittal^1^, Jack Scanlan^1^, Leigh Donnellan^1^, Manuela Klingler-Hoffmann^1^, Peter Hoffmann^1*^

^1^School of Pharmacy and Biomedical Science, College of Health, Adelaide University, Adelaide, SA 5000, Australia

^*^Corresponding author: Peter Hoffmann [peter.hoffmann@adelaide.edu.au](mailto:peter.hoffmann@unisa.edu.au)

ORCID

Darren Lau: 0000-0001-7501-6152

Parul Mittal: 0000-0003-0139-9757

Jack Scanlan: 0009-0007-9525-0609

Leigh Donnellan: 0000-0002-0042-2065

Manuela Klingler-Hoffmann: 0000-0003-2165-7813

^*^Peter Hoffmann: 0000-0001-8493-2208

**Table S1** Instrument settings for metabolite (small molecules and lipids) MALDI-MSI of oat tissue sections.

| Instrument settings | Small molecule | Lipid |
| --- | --- | --- |
| Mass range (*m/z*) | 50 to 700 | 300 to 1300 |
| Collision energy (eV) | 10 | 10 |
| Collision RF (Vpp) | 500 | 2500 |
| Ion energy (eV) | 5 | 15 |
| Transfer time (µs) | 45 | 80 |
| Pre pulse storage (µs) | 5 | 10 |

**Fig. S1** Further unsupervised segmentation of MALDI-MSI data

**Table S2** Metabolite identifications tentatively annotated using METASPACE.

| Molecule names | Adduct | *m/z* | Mass error (ppm) |
| --- | --- | --- | --- |
| 1,25-Dihydroxyvitamin D3-26,23-lactone | M+K | 483.2507 | 0.14378 |
| Eicosenoic acid | M+H | 311.2945 | -1.55332 |
| 2-Decaprenyl-6-methoxy-3-methyl-1,4-benzoquinone | M+Na | 855.6626 | -0.70383 |
| 3-Hydroxy-9-hexadecenoylcarnitine | M+H | 414.3214 | -1.05677 |
| 3-hydroxyhexadecanoyl carnitine | M+H | 416.337 | 1.746848 |
| Eicosatrienoic acid | M+H | 307.2632 | 0.243629 |
| 9(S)-HPODE | M+Na | 335.2193 | -0.45957 |
| All-trans-heptaprenyl diphosphate | M+Na | 677.3706 | 0.902508 |
| Choline | M+H | 104.107 | -1.48877 |
| Coenzyme Q10 | M+Na | 885.6731 | -0.92582 |
| DG 32:0 | M+Na | 591.4959 | 1.431942 |
| DG 34:1 | M+Na | 617.5115 | -1.63419 |
|  | M+K | 633.4855 | -1.01371 |
| DG 34:2 | M+Na | 615.4959 | 0.684483 |
| DG 36:2 | M+K | 659.5011 | -0.19546 |
| DG 36:3 | M+Na | 641.5115 | -1.19248 |
|  | M+K | 657.4855 | -1.71936 |
| DG 36:4 | M+Na | 639.4959 | 0.847249 |
| Docosa-4,7,10,13,16-pentaenoyl carnitine | M+Na | 496.3397 | 0.123636 |
| Eicosadienoic acid | M+H | 309.2788 | -1.84093 |
| Glycerophosphocholine | M+Na | 280.092 | -1.83511 |
| Hexacosanoic acid | M+Na | 419.386 | 0.24823 |
| Isoleucylproline, Leucylproline | M+H | 229.1547 | -0.52031 |
| L-Methionine | M+Na | 172.0403 | -1.56011 |
| LPA 16:0 | M+Na | 433.2326 | -1.26509 |
| LPA 18:2 | M+Na | 457.2326 | -0.41903 |
| LPA 20:1 | M+Na | 487.2795 | -1.29904 |
| LPA 20:3 | M+Na | 483.2482 | 1.193589 |
| LPA 20:4 | M+H | 459.2506 | -0.19381 |
| LPC 16:0 | M+H | 496.3398 | 0.051472 |
|  | M+Na | 518.3217 | -0.38414 |
|  | M+K | 534.2956 | -0.23683 |
| LPC 18:0 | M+H | 524.3711 | 1.175291 |
|  | M+Na | 546.353 | 0.325812 |
| LPC 18:1 | M+H | 522.3554 | -1.81019 |
|  | M+Na | 544.3374 | -1.61575 |
|  | M+K | 560.3113 | -1.2702 |
| LPC 18:2 | M+H | 520.3398 | -1.29984 |
|  | M+Na | 542.3217 | 1.32102 |
|  | M+K | 558.2956 | 0.358784 |
| LPC 18:3 | M+H | 518.3241 | -0.07899 |
| LPC 20:1 | M+Na | 572.3687 | 0.568975 |
| LPC 20:4 | M+H | 544.3398 | -2.83449 |
| LPC 20:5 | M+H | 542.3241 | -2.43747 |
| LPE 18:2 | M+H | 478.2928 | -1.51212 |
| LPE 20:0 | M+H | 510.3554 | -0.896 |
| LPE 22:2 | M+H | 534.3554 | 0.172238 |
| Maltotetraose | M+Na | 689.2111 | -0.62204 |
|  | M+K | 705.185 | 0.634391 |
| Maltotriose | M+Na | 527.1583 | 0.668783 |
|  | M+K | 543.1322 | -0.47567 |
| Octadecanamide | M+Na | 306.2767 | 1.949214 |
| Octadecenoic acid | M+H | 283.2632 | 0.017725 |
| PA 34:1 | M+Na | 697.4779 | 0.009575 |
| PA 36:2 | M+Na | 723.4935 | -0.86903 |
|  | M+K | 739.4675 | 0.471385 |
| PA 36:3 | M+Na | 721.4779 | -0.92131 |
|  | M+K | 737.4518 | 0.010071 |
| PA 36:5 | M+Na | 717.4466 | -2.65215 |
| PA 38:3 | M+Na | 749.5092 | -1.56302 |
|  | M+K | 765.4831 | -1.491 |
| PA 38:4 | M+Na | 747.4935 | 0.057057 |
|  | M+K | 763.4675 | 0.136788 |
| PA 38:5 | M+Na | 745.4779 | 0.25458 |
|  | M+K | 761.4518 | -1.19259 |
| PA 38:6 | M+Na | 743.4622 | -2.14907 |
|  | M+K | 759.4362 | -0.60017 |
| PA 38:7 | M+Na | 741.4466 | -2.23771 |
|  | M+K | 757.4205 | -2.61726 |
| PA 40:7 | M+Na | 769.4779 | -1.57773 |
| PA 40:6 | M+Na | 771.4935 | -1.28587 |
| p-Aminobenzoic acid | M+Na | 160.0369 | -0.83655 |
| PC 32:0 | M+H | 734.5694 | 1.252802 |
|  | M+Na | 756.5514 | 0.233711 |
| PC 34:0 | M+Na | 784.5827 | -1.3166 |
| PC 34:1 | M+H | 760.5851 | -0.6047 |
|  | M+Na | 782.567 | 1.051842 |
|  | M+K | 798.541 | -0.72623 |
| PC 34:2 | M+H | 758.5694 | 1.374087 |
|  | M+Na | 780.5514 | 0.070135 |
|  | M+K | 796.5253 | 1.67883 |
| PC 34:3 | M+Na | 778.5357 | 0.729689 |
| PC 36:1 | M+Na | 810.5983 | -2.11471 |
| PC 36:2 | M+H | 786.6007 | -0.21288 |
|  | M+Na | 808.5827 | -1.81426 |
|  | M+K | 824.5566 | -0.28958 |
| PC 36:3 | M+Na | 806.567 | -1.47666 |
|  | M+K | 822.541 | 0.1502 |
| PC 36:4 | M+H | 778.5381 | 0.630146 |
|  | M+Na | 804.5514 | 0.750804 |
|  | M+K | 820.5253 | 0.737099 |
| PC 36:5 | M+Na | 802.5357 | -0.81319 |
|  | M+K | 818.5097 | 0.322124 |
| PC 38:5 | M+H | 808.5851 | -0.40255 |
| PE 38:0 | M+Na | 798.5983 | 0.622454 |
| PE 38:1 | M+H | 774.6007 | -0.42826 |
| PE 38:6 | M+Na | 786.5044 | -2.70944 |
| PE 38:7 | M+Na | 784.4888 | -1.66968 |
|  | M+K | 800.4627 | 0.575822 |
| PE 40:6 | M+Na | 814.5357 | -1.42381 |
| PG 36:1 | M+Na | 799.546 | 1.579717 |
| PG 36:6 | M+Na | 789.4677 | 1.681311 |
| Phosphorylcholine | M+H | 184.0733 | -1.78916 |
| PI 34:2 | M+Na | 857.5151 | 1.712596 |
| PI 36:4 | M+Na | 881.5151 | -1.72674 |
|  | M+K | 897.489 | -0.53906 |
| Proline | M+H | 116.0707 | -2.58463 |
| Pyro-L-glutaminyl-L-glutamine | M+Na | 280.0904 | -0.94454 |
| Sucrose | M+Na | 365.1054 | 1.04234 |
|  | M+K | 381.0794 | -0.60103 |
| TG 50:1 | M+Na | 855.7412 | -0.2113 |
|  | M+K | 871.7151 | -0.19488 |
| TG 50:2 | M+Na | 853.7256 | 0.646022 |
|  | M+K | 869.6995 | 1.026674 |
| TG 52:2 | M+Na | 881.7569 | -0.9542 |
|  | M+K | 897.7308 | 0.083432 |
| TG 52:3 | M+Na | 879.7412 | 0.039031 |
|  | M+K | 895.7151 | 1.008073 |
| TG 52:4 | M+K | 893.6995 | 0.754553 |
| TG 52:5 | M+Na | 875.7099 | 0.519029 |
| TG 54:2 | M+Na | 909.7882 | -1.5166 |
| TG 54:3 | M+Na | 907.7725 | 1.466034 |
|  | M+K | 923.7464 | -0.45107 |
| TG 54:4 | M+Na | 905.7569 | -0.59199 |
|  | M+K | 921.7308 | 0.882517 |
| TG 54:5 | M+Na | 903.7412 | 0.481797 |
|  | M+K | 919.7151 | 1.303225 |
| TG 54:6 | M+Na | 901.7256 | 0.070136 |
|  | M+K | 917.6995 | 0.269258 |
| TG 56:3 | M+Na | 935.8038 | -0.58811 |
|  | M+K | 951.7777 | -1.34699 |
| TG 56:4 | M+K | 949.7621 | -1.32345 |
| Thymidine 5'-triphosphate | M+H | 482.9965 | -2.16576 |
| Valine | M+Na | 140.0682 | -2.14181 |
